# The costs in provision of haemodialysis in a developing country: A multi-centered study

**DOI:** 10.1186/1471-2369-12-42

**Published:** 2011-09-06

**Authors:** Priyanga Ranasinghe, Yashasvi S Perera, Mohamed FM Makarim, Aruna Wijesinghe, Kamani Wanigasuriya

**Affiliations:** 1 University Medical Unit, Colombo South Teaching Hospital, Kalubowila, Sri Lanka; 2 National Hospital of Sri Lanka, Colombo, Sri Lanka; 3 Kandy Teaching Hospital, Kandy, Sri Lanka; 4 Department of Clinical Medicine, Faculty of Medical Sciences, University of Sri Jayewardenepura, Sri Lanka

**Keywords:** haemodialysis, costs, developing country, Sri Lanka, renal failure

## Abstract

**Background:**

Chronic Kidney Disease is a major public health problem worldwide with enormous cost burdens on health care systems in developing countries. We aimed to provide a detailed analysis of the processes and costs of haemodialysis in Sri Lanka and provide a framework for modeling similar financial audits.

**Methods:**

This prospective study was conducted at haemodialysis units of three public and two private hospitals in Sri Lanka for two months in June and July 2010. Cost of drugs and consumables for the three public hospitals were obtained from the price list issued by the Medical Supplies Division of the Department of Health Services, while for the two private hospitals they were obtained from financial departments of the respective hospitals. Staff wages were obtained from the hospital chief accountant/chief financial officers. The cost of electricity and water per month was calculated directly with the assistance of expert engineers. An apportion was done from the total hospital costs of administration, cleaning services, security, waste disposal and, laundry and sterilization for each unit.

**Results:**

The total number of dialysis sessions (hours) at the five hospitals for June and July were 3341 (12959) and 3386 (13301) respectively. Drug and consumables costs accounted for 70.4-84.9% of the total costs, followed by the wages of the nursing staff at each unit (7.8-19.7%). The mean cost of a dialysis session in Sri Lanka was LKR 6,377 (US$ 56). The annual cost of haemodialysis for a patient with chronic renal failure undergoing 2-3 dialysis session of four hours duration per week was LKR 663,208-994,812 (US$ 5,869-8,804). At one hospital where facilities are available for the re-use of dialyzers (although not done during study period) the cost of consumables would have come down from LKR 5,940,705 to LKR 3,368,785 (43% reduction) if the method was adopted, reducing costs of haemodialysis per hour from LKR 1,327 at present to LKR 892 (33% reduction).

**Conclusions:**

This multi-centered study demonstrated that the costs of haemodialysis in a developing country remained significantly lower compared to developed countries. However, it still places a significant burden on the health care sector, whilst possibility of further cost reduction exists.

## Background

Chronic Kidney Disease (CKD) is defined as either renal damage or decreased renal function for three or more months, and is characterized by progressive destruction of renal mass with irreversible sclerosis and loss of nephrons [1]. CKD requiring renal replacement therapy is known as End-Stage Renal Disease (ESRD) [2]. Haemodialysis is one form of renal replacement therapy available for such patients and for patients with Acute Renal Failure (ARF) where dialysis is provided temporarily till the recovery of renal function. CKD is becoming a major public health problem worldwide, the average annual cost for maintenance of ESRD therapy excluding kidney transplantation was between US$ 70-75 billion worldwide in 2001, and the predicted number of ESRD patients will reach over 2 million by 2010 [3]. This enormous cost of treatment leads to a large burden on health care systems, particularly in developing countries like Sri Lanka.

In Sri Lanka, increasing attention has been focused on CKD and its treatment recently due to the rapid increase in prevalence. CKD of unknown etiology (CKDu) prevalent in North-Central Province of the country, while diabetes mellitus and hypertension are among the leading causes identified elsewhere in the country [4]. The total number affected by CKDu is unknown, however estimates suggests that in excess of 6000 people were undergoing treatment for this condition in 2005 [5]. In 2005, Anuradhapura Teaching Hospital alone recorded 742 live discharges and 140 deaths due to CKDu [5]. Mortality due to genitourinary diseases was the leading cause of death in many districts, being the 11th leading cause of Mortality in the country. In 2005, about 350 million rupees (4.6% of the Annual Health Budget) was spent on the management of patients with Renal Diseases [5]. Haemodialysis is the main form of renal replacement therapy available to patients with ESRD in Sri Lanka. This is offered free of charge at public sector hospitals funded by the government of Sri Lanka and at several private sector owned hospitals, where the costs are borne by the patients. The increasing prevalence of ESRD has resulted in the demands for haemodialysis far exceeding the service capacity of both public and private sector health care services of Sri Lanka. In view of this steady increase in the rate of entry of new patients into haemodialysis programs, it is necessary to adopt measures aimed at making the delivery of haemodialysis more efficient and cost effective [6,7]. To achieve this objective it is essential that the economic resources targeted towards this area be scientifically reviewed.

The present era of health resource management reinforces the longstanding need to make the best use of available resources. Hence management and financial data are being used to reshape health services worldwide and financial audits form a corner stone of these efforts. However, results of such audits are often criticized as being inaccurate, specific to an individual practices, difficult to understand and reproduce. This most often occurs as a result of the lack of knowledge and understanding of proper methodologies involved in conducting such financial audits among clinicians. There are no studies from Sri Lanka examining the processes and costs involved in provision of haemodialysis. The present study aims to provide a detailed analysis of the processes and costs of haemodialysis at public and private sector hospitals in Sri Lanka. In addition, we intend to provide a framework that could be utilized by clinicians for modeling similar financial audits.

## Methods

### Study setting

This prospective study was conducted at Haemodialysis units of three public (government) hospitals and two private hospitals in Sri Lanka for a period of two months in June-July 2010. The three public hospitals were; 1) The National Hospital of Sri Lanka (NHSL), 2) Colombo South Teaching Hospital (CSTH) and 3) The Kandy Teaching Hospital (KTH). The NHSL is the largest hospital in Sri Lanka with a bed strength of nearly 3300, while the KTH (the second largest hospital) and CSTH has bed strengths of nearly 2300 and 1100 respectively [8]. The two private sector hospitals identified as Hospital 'A' and Hospital 'B' had bed strengths of nearly 250 each. Permission to conduct the study was obtained from the medical directors of the respective hospitals. The study conformed to the Helsinki Declaration and was conducted in accordance with local legislation. Ethical approval for the study was obtained from the Ethics Review Committee of the Colombo South Teaching Hospital, Kalubowila, Sri Lanka.

### Data collection and analysis

Data for two consecutive months (June and July 2010) were collected from the five dialysis units. A review of audit data of each dialysis unit showed that the workload during the study period was similar with that of previous months. Some data were collected prospectively and others were obtained retrospectively from perusal of records maintained by the nursing staff. Initially two members of the study team visited each dialysis unit separately and performed an independent assessment for a period of two weeks on the process of dialysis and types of costs involved at each unit. This primary data were analyzed and the costs items involved under the categories of a) direct costs and b) indirect costs were identified (Table 1). Workload, patient information and consumables used were collected from the registries maintained in the respective units.

**Table 1:** Cost items and categories associated with provision of haemodialysis

Cost item	Cost category	Cost type
1. Administration	Indirect cost	Variable indirect
2. Cleaning services	Indirect cost	Variable indirect
3. Drugs and consumables	Direct cost	Variable direct
4. Electricity	Direct cost	Variable direct
5. Investigations	Direct cost	Variable direct
6. Laundry and sterilization	Direct cost	Variable direct
7. Maintenance (Building and Equipment)	Direct cost	Fixed direct
8. Security	Indirect cost	Variable indirect
9. Staff wages	Direct cost	Variable direct
a. Doctors		
b. Nurses		
c. Minor staff (attendants and labourers)		
10. Waste disposal	Indirect cost	Variable indirect
11. Water	Direct cost	Variable direct

Cost of the drugs and consumables for the three public hospitals were obtained from the price list issued by the Medical Supplies Division of the Department of Health Services [9], while for the two private hospitals they were obtained from the respective financial departments. Staff wages were obtained from the chief accountant/chief financial officer of each hospital. The cost of electricity per month for each dialysis unit was calculated with the assistance of an engineer from Ceylon Electricity Board (CEB). The total Watt consumption per month at each dialysis unit was calculated taking into account all electrical appliances at the unit, Watts consumed by each appliance, duration of usage of appliances per day and number of days the unit operates per month. The monthly electricity consumption thus calculated was then used for calculation of the monthly electricity cost according to standard CEB rates.

The cost of water was calculated by a similar direct method with the assistance of an engineer from Ceylon Water Board (CWB). The total monthly water consumption at each dialysis unit was calculated by multiplying total monthly hours of dialysis by the hourly water usage per dialysis machines (as specified in the product information leaflet) and number of machines at each dialysis unit. This was then used for calculation of monthly water costs according to standard CWB rates. The usage of water for other purposes at the dialysis units were negligible in comparison and were excluded.

The total hospital costs of administration, cleaning services, security, waste disposal and, laundry and sterilization were obtained from the chief accountant/chief financial officer at each hospital. An apportion was done from this total cost to obtain cost for each dialysis unit based on the following method,

1. Cost of administration, cleaning services and security - percentage surface area of the dialysis unit in comparison to the total hospital surface area.

2. Waste disposal and, laundry and sterilization - percentage monthly patient turnover at the dialysis unit in comparison to the total hospital monthly patient turnover.

The aim of the present study was to calculate the operational costs of a haemodialysis unit thus capital expenses, such as expenditure for buildings and furniture were not included. A few recurrent expenditures, such as maintenance, transport and mortuary costs were negligible and were also not included in the calculation. All data were double-entered and cross checked for consistency. Data were analysed using SPSS version 14 (SPSS Inc., Chicago, IL, USA) statistical software package.

## Results

The total number of dialysis sessions at the five hospitals for the months of June and July 2010 were 3341 and 3386 respectively, while the total hours of dialysis were 12959 and 13301. The indication for a majority of the dialysis were Chronic Renal Failure (June - 82.1%, July - 81.2%). A summary of number dialysis, hours and dialysis unit characteristics are provided in Table 2.

**Table 2:** Total number of dialysis sessions, dialysis hours & unit characteristics at each hospital

	NHSL	KTH	CSTH	Hospital 'A'	Hospital 'B'	Total
Dialysis sessions						
June	562	1561	73	443	702	3341
July	668	1530	73	440	675	3386

Hours of dialysis						
June	2094	6152	230	1772	2711	12959
July	2534	5946	240	1760	2821	13301

Dialysis machines	10	28	2	6	8	54

Hours of dialysis per machine						
June	209.4	219.7	115	295.3	338.9	240.0
July	253.4	212.4	120	293.3	352.6	246.3

Doctors	0	2	0	0	4	6

Nursing staff	19	48	5	9	12	93

"Minor" staff	8	10	1	4	2	25

ARF - Acute Renal Failure, ESRD - End Stage Renal Disease, NHSL - The National Hospital of Sri Lanka, KTH - Kandy Teaching Hospital, CSTH - Colombo South Teaching Hospital

The percentage surface area of the dialysis units at NHSL, KTH, CSTH, Hospital 'A' and Hospital 'B' were 0.06%, 0.45%, 0.164%, 0.5%, 1.0% respectively, while the percentage patient turnover was 0.77%, 1.0%, 0.3%, 1.0%, 1.5% respectively. The breakdown of the costs of haemodialysis given as the average for the months of June and July at each hospital is given in Table 3. Drug and consumables costs accounted for 70.4% to 84.9% of the total costs (NHSL - 78.9%, KTH - 74.0%, CSTH - 70.4%, Hospital 'A' - 84.9%, Hospital 'B' - 79.7%), followed by the wages of the nursing staff at each unit (7.8% to 19.7%). Both renal and non-renal nurses were attached to the respective dialysis units, however there was no difference in salary between the two group. The percentage contribution to the total costs by each of the other cost items was less than 4.0%.

**Table 3:** Costs of haemodialysis at each hospital

	Average costs for June and July - LKR (US$)*				
	
	NHSL	KTH	CSTH	Hospital 'A'	Hospital 'B'
Administration	139,734 (1,236)	52,213 (462)	3,936 (35)	32,156 (284)	138,909 (1,229)

Cleaning services	7,739 (68)	12,212 (108)	2,952 (26)	5,500 (49)	17,708 (157)

Drugs and consumables	3,846,883 (34,043)	5,940,705 (52,573)	483,474 (4,278)	2,536,491 (22,447)	3,474,731 (30,750)

Electricity	153,214 (1,356)	364,425 (3,225)	26,150 (231)	82,220 (728)	132,192 (1,170)

Laundry and sterilization	9,300 (82)	27,900 (247)	6,600 (58)	6,000 (53)	7,000 (62)

Security	1,336 (12)	4,883 (43)	1,148 (10)	9,500 (84)	17,039 (151)

Staff wages					
Doctors	0 (0)	66,000 (584)	0 (0)	0 (0)	169,486 (1,500)
Nurses	513,000 (4,540)	1,296,000 (11,469)	135,000 (1,195)	234,000 (2,071)	367,113 (3,249)
Minor staff	192,000 (1,699)	240,000 (2,124)	24,000 (212)	62,400 (552)	14,940 (132)

Waste disposal	1,700 (15)	5,100 (45)	2,100 (18)	14,000 (124)	10,000 (88)

Water	7,512 (66)	18,147 (161)	910 (8)	5,298 (47)	9,770 (86)

Total	4,872,418 (43,119)	8,027,585 (71,041)	686,270 (6,073)	2,987,565 (26,439)	4,358,888 (38,574)

*1 US$ = Sri Lankan Rupees (LKR) 113, NHSL - The National Hospital of Sri Lanka, KTH - Kandy Teaching Hospital, CSTH - Colombo South Teaching Hospital

The total cost per month for haemodialysis at the five hospitals combined was LKR 22,491,384 (US$ 199,038), hence the annual haemodialysis cost at these five hospitals alone is nearly LKR 0.27 billion. The cost of haemodialysis per hour at NHSL, KTH and CSTH were LKR 2,105 LKR 1,327 and LKR 2,923 respectively. Hospital 'A' and Hospital 'B' each charged their patients LKR 1,750 for an hour of haemodialysis. The actual cost of haemodialysis per hour at Hospital 'A' and Hospital 'B' were LKR 1,692 and LKR 1,575 respectively. The costs of a four hour haemodialysis session at NHSL, KTH, CSTH, Hospital 'A' and Hospital 'B' were LKR 8,420, LKR 5,308, LKR 11,692, LKR 6,768 and LKR 6,300 respectively.

Patients with ESRD in Sri Lanka usually undergo 2 to 3 haemodialysis sessions of four hours per week. The mean cost of a four hour dialysis session in Sri Lanka per patient was LKR 6,377 (US$ 56). The annual cost of haemodialysis for a patient with chronic renal failure undergoing 2-3 dialysis session of four hours duration per week was LKR 663,208 - 994,812 (US$ 5,869 - 8,804).

At KTH facilities are available for the re-use of dialyzers, although this was not available during study period due to logistical problems. However, when this programme was active the dialyzers were re-used for five dialysis sessions after sterilization. If this was carried out during the study period the cost of consumables at KTH would have come down from LKR 5,940,705 to LKR 3,368,785 (43% reduction). This would reduce costs of haemodialysis per hour from LKR 1,327 at present to LKR 892 (33% reduction).

## Discussion

The results of this study demonstrates that the cost of haemodialysis in Sri Lanka is higher than in India [10] and Sudan [11], but remained considerably less than that of Brazil [12], Iran [13], Barbados [14], France [15], Japan [16], Canada [17] and USA [18] (Table 4). The differences noted in the reported cost in different studies are very high and cannot be explained solely in terms of their annual per capita income between the countries. A combination of factors play a role including different management protocols, in-patient care, different local labor costs, import duties, etc, for example, while drugs and consumables cost constituted 70-85% of overall expenses of dialysis in Sri Lanka, in Iran and Greece it accounted for 12% and 53% of the cost respectively [13,19]. In addition, the data on haemodialysis costs are mostly from western developed countries, while there is a relative lack of data from developing countries and the Asian region. 4). However, since the aim of the present study was to calculate the operational costs of a haemodialysis unit thus capital expenses, such as expenditure for buildings and furniture were not included. This needs to be borne in mind when comparing our finding with that of other countries that have included these capital expenses.

**Table 4:** Comparison of total haemodialysis cost per patient between countries

Country, year [Reference]	Annual expenses per patient (US$)	Cost items included
India, 2009 [10]	$ 3,000	3, 4, 7, 9, 11, 10
Sri Lanka, 2010	$ 5,869 - 8,804	1, 2, 3, 4, 5, 6, 7, 8, 9
Sudan, 2010 [11]	$ 6,847	3,7, 10, 12
Brazil, 2007 [12]	$ 7,980 - 13,428	1, 2, 3, 4, 5, 7, 8, 9, 12
Iran, 2009 [13]	$ 11,549	1,2, 3, 4, 7, 8, 9, 10, 11, 12, 13,
Barbados, 2004 [14]	$ 17,029	1, 2, 3, 4, 5, 7, 9, 10
France [15]	$ 34,452 - 39,089	1, 2, 4, 5, 7, 8, 9, 10, 12, 13
Japan, 2001 [16]	$ 46,000	1, 2, 3, 4, 5, 6, 7, 8, 9, 12
Canada, 2002 [17]	$ 42,057 - 51,252	1, 2, 3, 4, 5, 7, 9, 10, 12
USA, 2010 [18]	$ 77,506	1, 2, 3, 4, 5, 7, 8, 9, 10, 11, 12

1- Administration; 2- Cleaning services; 3- Drugs and consumables; 4- Electricity; 5- Laundry and sterilization; 6- Security; 7- Staff wages; 8- Waste disposal; 9- Water; 10- Capital expenses (buildings, machines, instruments, etc); 11- Maintenance and repair; 12- Hospitalization costs; 13- Personal costs to patients;

In a country where the annual per capita income is US$ 2,029 [20], comparatively the annual cost of dialysis of US$ 5,869 - 8,804 is significantly high. This and the increasing prevalence of ESRD place a significant burden economically on the health care sector in Sri Lanka. Hence, there is a timely necessity for cost reduction methods. In the long term, the most important factor to reduce the overall annual cost is to reduce the number of patients with ESRD. This goal can be achieved by preventing the progression of renal disease and implementing strategies for early detection and optimal treatment of causative diseases.

The main contributor to cost in Sri Lanka was the cost of drugs and consumables, thus strategies aimed at reducing these costs would help to reduce annual cost in the short term. One such method is the reuse of the dialyzers implemented by KTH, which would successfully reduced the costs by nearly 35%. Reuse provides a significant economic benefit, however recent reports documenting certain pitfalls such as increased morbidity and mortality and disease transmission, with reduced dialyzer efficiency have cast doubts on the success of this strategy [21].

**Figure 1:**
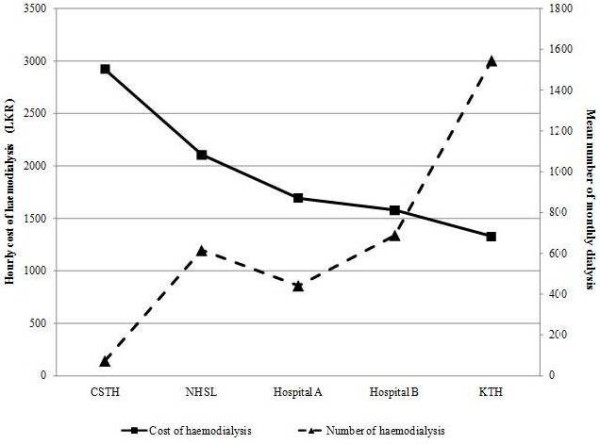
**The mean number of haemodialysis sessions per month and dialysis cost per hour at each hospital**.

The hourly cost of haemodialysis at each hospital was inversely proportionate to the mean number of monthly dialysis (Figure 1). The hourly cost of dialysis was highest at CSTH and lowest at KTH, with the lowest and highest mean number of dialysis per month respectively. The haemodialysis unit at CSTH mainly serves the purpose of dialyzing in hospital patients with ARF or Acute on Chronic Renal Failure while also handling transferred patients from nearby smaller hospitals. These smaller units elsewhere in the country helps to minimize the workload of larger dialysis centers such as NHSL and KTH, while improving the outcome of patients with ARF by minimizing delays associated with transfers. Thus the resources at these smaller haemodialysis units may be underutilized with respect to CKD patients resulting in the overall costs being higher in comparison to larger haemodialysis units. However the increased workload at larger hospitals such as NHSL and KTH has resulted in a dialysis machine operating in excess of 200 hours per month (Table 2). This may increase costs associated with machine maintenance and breakdown repair while in addition contributing to reduced efficiency of haemodialysis.

Studies conducted in other countries have demonstrated that kidney transplantation is the most cost-effective treatment for end stage renal disease, offering considerable savings and a drastic improvement in quality of life in these patients [22,23]. Considering the fact that haemodialysis is the most common renal replacement therapy for patients in Sri Lanka, it is highly recommended that we try to improve our kidney transplant programmes. Annual cost of Chronic ambulatory peritoneal dialysis (CAPD) per patient is similar to the cost of haemodialysis but is done infrequently as dialysis fluid in not freely available. Patients' perception on CAPD is largely unknown. Therefore, further studies are required in Sri Lanka, comparing the costs of the different renal replacement therapy modalities.

This study has several limitations. We did not consider costs which end stage renal disease imposes on society in terms of production losses due to treatment requirements, morbidity, mortality, and time spent to care for patients. In addition personnel costs incurred by patients were also not included in the study. The lack of data from all renal replacement therapy providers in Sri Lanka prevented us from estimating the overall disease burden caused by ESRD to the economy. Erythropoietin and intravenous iron supplementation is frequently offered to some patients with CKD during dialysis in Sri Lanka. However, these were not offered to all patients undergoing haemodialysis and thus were excluded from the cost of consumables at each hospital.

## Conclusion

This multi-centered study demonstrated that the costs of haemodialysis in a developing country remained significantly lower compared to developed countries. However, it still places a significant burden on the health care sector, whilst possibility of further cost reduction exists.

## List of Abbreviations

ARF: Acute Renal Failure; CEB: Ceylon Electricity Board; CKD: Chronic Kidney Disease; CSTH: Colombo Teaching Hospital; CWB: Ceylon Water Board; ESRD: End Stage Renal Disease; KTH: Kandy Teaching Hospital; LKR: Sri Lankan Rupees; NHSL: National Hospital of Sri Lanka

## Competing interests

The authors declare that they have no competing interests

## Authors' contributions

PR, MFMM and KYSP made substantial contribution to conception and study design. PR, MFMM, KYSP and AW were involved in data collection. PR, KW and KYSP were involved in refining the study design, statistical analysis and drafting the manuscript. KW, MFMM and AW critically revised the manuscript. All authors read and approved the final manuscript.

## Pre-publication history

The pre-publication history for this paper can be accessed here:

http://www.biomedcentral.com/1471-2369/12/42/prepub
